# The trends of human dirofilariasis in Croatia: Yesterday – Today – Tomorrow

**DOI:** 10.1016/j.onehlt.2020.100153

**Published:** 2020-07-21

**Authors:** Ana Pupić-Bakrač, Jure Pupić-Bakrač, Daria Jurković, Maja Capar, Lorena Lazarić Stefanović, Izabela Antunović Ćelović, Jasmina Kučinar, Adam Polkinghorne, Relja Beck

**Affiliations:** a Department of Ophthalmology, General Hospital Zadar, Bože Peričića 5, 23 000, Zadar, Croatia; b Department of Otorhinolaryngology and Maxillofacial Surgery, General Hospital Zadar, Bože Peričića 5, 23 000, Zadar, Croatia; c Department for Bacteriology and Parasitology, Croatian Veterinary Institute, Savska cesta 143, 10 000 Zagreb, Croatia; d Department of Infectology, General Hospital Pula, Zagrebačka 30, 52100, Pula, Croatia; e Department of Microbiology, General Hospital Pula, Zagrebačka 30, 52100, Pula, Croatia; f Microbiology Department, Istria County Institute of Public Health, Nazorova ulica 23, 52100 Pula, Croatia; g Department of Microbiology and Infectious Diseases, New South Wales Health Pathology, Nepean Blue Mountains Pathology Service, PO Box 63, Penrith, New South Wales, 2751, Australia

**Keywords:** Dirofilariasis, Humans, Eye, Skin, Helminths, Endemic diseases

## Abstract

**Introduction:**

Human dirofilariasis is a disease historically linked to the Mediterranean area. For the last few decades, however, *Dirofilaria* nematodes have been spreading, both in terms of prevalence and the geographical expansion in non-endemic areas. Currently, cases of human dirofilariasis are recorded in more than 40 countries worldwide. Croatia is considered an endemic area of the Adriatic basin.

**Methods:**

In a nationwide investigation, new and previously published cases of human dirofilariasis in Croatia were analyzed.

**Results:**

Since 1996, 30 cases of human dirofilariosis were reported in Croatia. A total of 14 (46,67%) cases were from the coastal and 16 (53,33%) from continental regions of the country. Based on anatomical location, 13 (43,33%) cases were subcutaneous, 12 (40%) were ocular and five (16,67%) occurred in the reproductive organs. In all 30 cases, *Dirofilaria repens* was identified as the causative agent.

**Conclusions:**

An increase in air temperature as climate change, changes in mosquito fauna, high prevalence of *D. repens* in dogs and limited use of chemoprophylaxis are possible risk factors for *Dirofilaria* infection in the Croatian population. Since reporting to epidemiological services is not mandatory in this country, the real number of human dirofilariasis cases is probably significantly higher than published. This emphasizes the need for mandatory reporting of human cases and surveillance of *Dirofilaria* infection in dogs and mosquitoes in Croatia, following the “One Health” concept.

## Highlights

• A total of 30 cases of human dirofilariasis have been described in Croatia.
• In all cases, *D. repens* was identified as the causative agent.
• Geographical distribution among regions was almost identical.
• Subcutaneous nodules were not the major clincal presentation of *D. repens* infections.

## 1 Introduction

Dirofilariasis is a helminthic infection caused by nematode parasites of the *Dirofilaria* genus, which are natural parasites of carnivorous animals, primarily domesticated dogs. There are more than 40 described species of *Dirofilaria*, but human infection is most commonly caused by two species – *Dirofilaria repens* and *Dirofilaria immitis* [1]. The vectors are females of various mosquito species from the *Anopheles, Aedes, Ochlerotatus, Culex, Culiseta* and *Coquillettidia* genera [2]. Because dirofilariasis is zoonotic and vector-borne, it is best understood from a One Health perspective.

Humans are considered to be accidental hosts in which *Dirofilaria* spp. rarely reach sexual maturity but induce local inflammation and/or granuloma formation [3]. *Dirofilaria immitis*, which occurs in the heart of dogs, typically infects the pulmonary blood vessels when it infects humans. In contrast, the traditional clinical picture of human dirofilariasis caused by *D. repens* is most frequently manifested with one of two clinical forms – subcutaneous and ocular, although cases of infection at various anatomic locations such as the lungs, oral cavity, cerebrum, testes, and female breast have been reported in the literature [4,5]. Subcutaneous forms present as a nodule in subcutaneous tissues, usually about one centimetre in size. In the majority of cases, symptoms are mild or unrecognized, and only sometimes larva migrans-like symptoms are present (i.e. irritation and itching) [5]. Subcutaneous nodules have been reported in various human body areas with a predilection for superficial tissues of the facial regions (perioral, periorbital, forehead), the skin of the lower leg, soft tissues of the hand or finger, subcutaneous tissue of the hypogastrium and the neck [4,6].

Ocular dirofilariasis mainly manifests in subconjunctival form, which is relatively easily diagnosed and surgically extracted. However, if not diagnosed in a timely manner, the parasite can migrate in the peri-, intra- or retro-ocular space, becoming a major diagnostic and therapeutic challenge [7,8]. Complications of the disease include damaged vision, floaters, glaucoma, retinal detachment, vitreous opacity, loss of visual acuity and even blindness [1,9].

Reporting of human dirofilariasis is not mandatory in Croatia despite the emergence of cases. In the recent review on *D. repens* infection in Europe, precise data referring to human cases in Croatia are scarce [5]. To cover that knowledge gap, in the current study we present new cases of human dirofilariasis and an overview of all published cases in Croatia.

## 2 Methods

### 2.1 Clinical case reports

**Case report 1.**

**Fig. 1:**
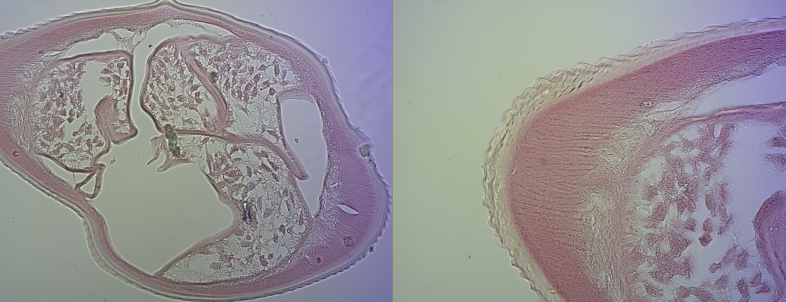
Hematoxylin and eosin–stained histological cross-section of the filarial worm from Case 1. Typical features of *Dirofilaria repens* are identified, including external longitudinal ridges, the thick cuticula, the well-developed musculature and the internal structure.

A 64-year-old male patient was examined due to the presence of firm, migrating nodules in the brachial region, without a history of any serious disease. No travel outside of Croatia was reported in the previous months; however, he described spending large amounts of recreational time in forests and islands near his residence in Smoljanci, Istria. He had a pet dog. On physical examination, a painless, well-demarcated, subcutaneous nodule was found in his right upper arm, 1.5 × 1.5 × 1.0 cm in size. The ultrasonographic finding of the nodule appeared normal. The total blood counts, erythrocyte sedimentation rate and C-reactive protein were within normal values. Serologic results for trichinosis, toxocariasis, schistosomiasis, distomatosis and cysticercosis were negative. Excision in local anaesthesia was proposed. During the operative procedure, a filarial worm was found, and based on histopathology, *D. repens* infection was suspected (Fig. 1). Symptoms resolved promptly after surgical removal of the nematode. The patient was followed for the next 12 months, and no additional symptoms were noted. The patient's dog was referred to veterinarian examination and canine dirofilariasis was excluded.

**Case report 2.**

**Fig. 2:**
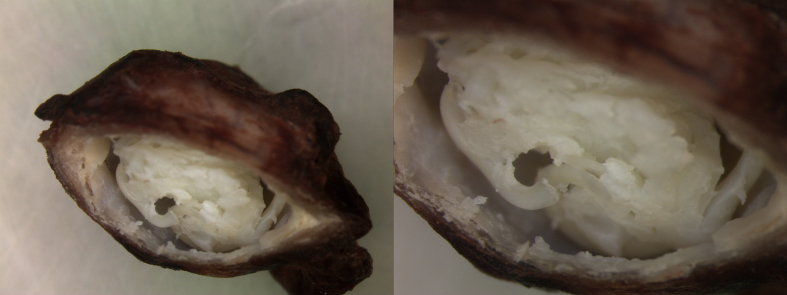
Surgical specimen from Case 2. Photograph showing filarial worm inside the surgically excised cyst. The dirofilariotic cyst was located in the upper eyelid.

A 72-year-old female patient from the Istrian region sought urgent ophthalmic examination for sudden swelling of the upper lid of the right eye. No information on potential injury was available, and she described no history of allergies. She had no pets. There was no history of travel outside Croatia. On examination, the lid was found to be edematous. Lid swelling was soft, cystic, and non-tender. Slit-lamp examination showed no significant congestion and chemosis of the conjunctiva, and the cornea was clear. The rest of the ocular examination was unremarkable. The ultrasonographic finding suggested cystic formation in the upper eyelid. Based on clinico-radiological findings, an epidermoid cyst was suspected. Surgical excision was performed under local anaesthesia and macroscopic examination revealed filarial worms (Fig. 2). Microscopic analysis suggested the diagnosis of *D. repens* infection. Symptoms resolved one day after surgical removal of parasite. Following extraction, the patient had no complications or recurrences.

**Case report 3.**

**Fig. 3:**
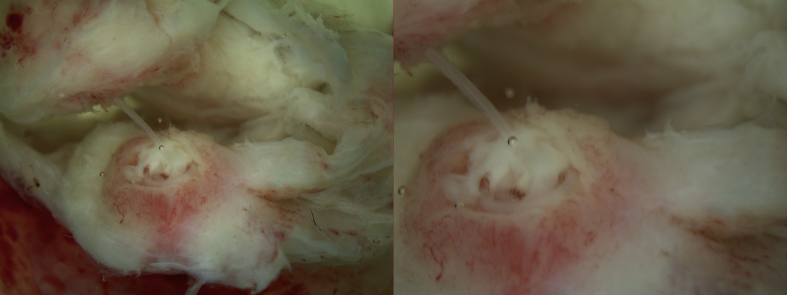
Surgical specimen from Case 3. Photograph showing cystic nodule containing a filarial worm surgically removed from subcutaneous tissue of the upper arm.

A 45-year-old male attended an outpatient examination for a nodule of the left upper arm. Ten days prior, the patient reported the appearance of a nodule on the left shoulder, accompanied with pain and redness. After three days, the shoulder nodule retreated and oedema of the left breast followed. Breast oedema was present for four days, after which a nodule of the upper arm appeared. From anamnesis, the patient indicated he lived in Pula and worked in Galižana, a small village in Istria. He had a pet cat and was in close proximity to the dogs in the workplace. He was exposed to mosquitoes during work and had multiple mosquito bites. On examination, a swelling of 1.7 × 1.4 × 0.7 cm in size was found in the distal third of the upper arm, without hyperemia and pain upon palpation. The total blood counts, erythrocyte sedimentation rate and C-reactive protein were within normal values. Subcutaneous dirofilariasis was suspected and excision with local anaesthesia proposed. During the operative procedure, a live nematode was found and removed together with surrounding subcutaneous tissue (Fig. 3). Symptoms resolved promptly after surgical extraction of the nematode. No complications or recurrences were noted during follow-up.

Nematodes were sent to the Croatian Veterinary Institute, Zagreb, Croatia (Department of Bacteriology and Parasitology) for molecular identification.

### 2.2 Molecular identification and genotyping

For species conformation, parasites or tissue samples were cut into pieces and DNA was extracted using the DNA ‘Blood and tissue kit’ (Qiagen, Hilden, Germany) in the automatic extraction system Qiacube (Qiagen, Hilden, Germany). Molecular identification of the parasite was conducted with forward DIDR-F1 (5′ – AGT GCG AAT TGC AGA CGC ATT GAG - 3′) and reverse DIDR-R1 (5′ – AGC GGG TAA TCA CGA CTG AGT TGA - 3′) primers and GoTaq® G2 Hot Start Colorless Master Mix (Promega). Conventional PCR Amplification was performed on ProFlex PCR System (Applied Biosystems, USA) according to the protocol of Rinshiwet al. (2006). The primers targeted the Internal Transcribed Spacer Region 2 (ITS2) for *D. immitis* (542 bp), *D. reconditum* (578 bp) and *D. repens* (484 bp). Species-specific PCRs amplifying a fragment of approximately 200 bp specific to the cytochrome oxidase subunit 1 (COI) for *D. immitis* DI COI —F1 AGT GTA GAG GGT CAG CCT GAG TTA and DI COI-R1 ACA GGC ACT GAC AAT ACC AAT and for *D. repens* DR COI-F1 AGT GTT GAT GGT CAA CCT GAA TTA and DR COI-R1 GCC AAA ACA GGA ACA GAT AAA ACT were also subsequently applied (Rinshiw et al. 2006). For sequencing, the protocol described by Casiraghi et al. (2001) was used to amplify a 667-bp region of the cytochrome *c* oxidase I gene. The amplified products were analyzed by capillary electrophoresis (QIAxcel System®, QIAGEN) with size markers in the bp ranges of 100–2500. Samples were purified with ExoSAP-IT® (USB Corp., Cleveland, United States) and sequenced in both directions in the commercial company (Macrogen Inc., Netherlands). Sequences were assembled using the SeqMan Pro software, edited with EditSeq of the Lasergene software (DNASTAR, Madison WI, USA) and compared with available sequences using BLAST.

### 2.3 Secondary data collection

Metadata on cases of human infection with *Dirofilaria* spp. in Croatia were analyzed. The studies and their analysis examined in the present work are the result of extensive text mining and searching through electronically available databases (Medline/PubMed, Web Of Science, Embase, Scopus, PsychInfo, CINAHL, Hrčak), individual journals, proceedings papers and meeting abstracts for all results retrieved by searches of any of the keywords: “Dirofilariasis”, “Human dirofilariasis”, “*Dirofilaria repens*“, “*Dirofilaria immitis*, “*Dirofilaria tenuis*”, “Animal dirofilariasis”, “Zoonosis”, “Vector-borne disease”, “Parasite”, “Helminths”, “Nematode”, “Mosquito”, “Dog”, “Croatia”, “Adriatic”, “Mediterranean”, “Europe”, “Endemic”; as well as their combinations. References to published articles were used to obtain additional articles. Both studies written in English and Croatian language were analyzed. After the screening of all identified articles, only those that met the criteria for eligibility were included in the study. The cross-reference list of the articles included in the review was manually checked for relevant studies. The review contained articles published until April 2020.

### 2.4 Statistical analysis

After obtaining necessary data, statistical analysis was conducted. SPSS for WINDOWS (version 13.0 SPSS Inc. Chicago, Illinos, USA) and Microsoft Excell (version of Office 2007, Microsoft Corporation, Redmont, WA, USA) were used. Nominal variables are represented as absolute (number) and relative (percentage) frequency.

## 3 Results

### 3.1 Characterization of *Dirofilaria* spp

In all three presented cases, 480 bp fragments of the ITS2 and gene fragments of COI were PCR amplified for *D. repens* but not for *D. immitis*. All three 680 bp partial COI gene sequences were identical to each other and, following BLAST comparison, were found to be identical to sequences of *D. repens* from the scrotum of a Croatian man (Genbank accession number KX265049).

### 3.2 Epidemiology and clinical characteristics

The bibliography search retrieved 16 articles reporting on 27 cases of human dirofilariasis in Croatia [[10], [11], [12], [13], [14], [15], [16], [17], [18], [19], [20], [21], [22], [23], [24], [25]]. The first published article was printed in 1996, and the last in 2019, with the highest rate of published cases in 2015 (*N* = 7). Out of all published articles, 10 were case report/series, five were conference proceedings and one was a letter to the editor. In addition to cases from the current study, 30 human cases were documented in total.

**Fig. 4:**
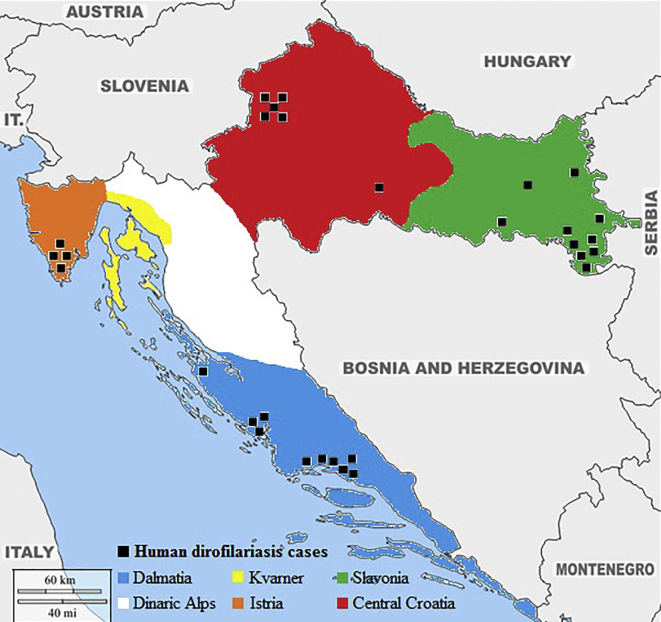
Geographic map showing distribution of *Dirofilaria repens* human infection in Croatia.

Reported cases included children and adults in the age range of 3–77 years. Geographical distribution among regions was almost identical; 14 (46,67%) cases were from the coastal and 16 (53,33%) from the continental region of Croatia (Fig. 4) (Table 1). A total of 13 (43,33%) cases had subcutaneous, 12 (40%) had ocular, four (13,33%) had genital and one (3,33%) had mammary form of dirofilariasis. Futhermore, among genital cases, the parasite was detected in the epididymis in two (6,67%) cases, the scrotum (1 case; 3,33%) and mons pubis (1 case; 3,33%). All 30 (100%) cases underwent surgical treatment (excision). In all 30 (100%) cases, *D. repens* was identified as the causative agent, based on morphological and/or molecular confirmation. Table 2 summarizes the epidemiological and clinical features of all reported cases of dirofilariasis in Croatia.

**Table 1:** Epidemiological characteristics including number of mosquito species, prevalence of dirofilariasis in dogs, reports/cases of human dirofilariasis, clinical forms and causative species of human dirofilariasis in coastal/continental/all Croatia. N – number; d. – dirofilariasis; Sc – subcutaneous; O – ocular; G – genital; M – mammary; *R* – *repens*; *I* – *immitis*.

Epidemiological characteristics	Coastal Croatia	Continental Croatia	All Croatia	References
Mosquito species (N)⁎	25–27	20–32	53	[[26], [27], [28], [29], [30], [31]]
Canine d. prevalence (%)	8–30.5	15.5–47.3	8–47.3	[[32], [33], [34], [35]]
Human d. reports (N)⁎⁎	7	10	17	[16,22,[10], [11], [12], [13], [14], [15],[17], [18], [19], [20], [21],[23], [24], [25]]
Human d. cases (N)	14	16	30	
Form Sc:O:G:M (N)	10:2:1:1	3:10:3	13:12:5	
Species *R*:*I* (N)	14:0	16:0	30:0	

⁎ Croatian mosquito fauna comprises eight genera: *Anopheles*, *Aedes*, *Ochlerotatus*, *Culex*, *Culiseta*, *Coquillettidia*, *Orthopodomyia* and *Uranotaenia*. The most abundant mosquito species in the coastal fauna are: *Culex pipiens complex*, *Ochlerotatus sticticus*, *Culex hortensis*, *Culiseta longiareolata* and *Culiseta annulata*. The most abundant mosquito species in the continental fauna are: *Culex pipiens complex*, *Ochlerotatus sticticus*, *Ochlerotatus cantans*, *Ochlerotatus geniculatus* and *Aedes vexans*.

⁎⁎ All published reports on human dirofilariasis in Croatia (*N* = 16) and the current study (N = 1).

**Table 2:** Epidemiological and clinical features of all reported cases of human dirofilariasis in Croatia. Ref – reference; No – number; Unknown – unspecified or data unavailable; *D*.– *Dilofilaria*.

Ref	Year	No of cases	Geographic Region	Traveling history	Animal contact	Form of infection	Anatomical site	*D.* species
[10]	1996	1	Central Croatia	International (Kosovo)	Unknown	Ocular	Subconjunctival	*D. repens*
[11]	2003	2	2 Dalmatia	1 International (France) 1 Unknown	2 Unknown	2 Subcutaneous	1 Head (temporal) 1 Elbow	*2 D. repens*
[12]	2005	5	5 Dalmatia	5 Unknown	5 Unknown	5 Subcutaneous	5 Unknown	*5 D. repens*
[13]	2006	1	Dalmatia	No	Unknown	Mammary	Mammary gland	*D. repens*
[14]	2007	1	Central Croatia	Unknown	Unknown	Ocular	Lacrimal gland	*D. repens*
[15]	2007	1	Dalmatia	Unknown	Unknown	Genital	Epididymis	*D. repens*
[16]	2007	2	2 Central Croatia	1 Domestic 1 No	1 Domestic animals 1 No	2 Subcutaneous	1 Lumbal region 1 Thigh	*2 D. repens*
[17]	2008	1	Central Croatia	Domestic	Dog	Subcutaneous	Thigh	*D. repens*
[18]	2010	1	Slavonia	Unknown	Dog and cat	Ocular	Subconjunctival	*D. repens*
[19]	2013	1	Istria	Unknown	Unknown	Subcutaneous	Forearm	*D. repens*
[20]	2013	1	Central Croatia	No	Dog and cat	Ocular	Subconjunctival	*D. repens*
[21]	2015	1	Slavonia	Unknown	Unknown	Genital	Epididymis	*D. repens*
[22]	2015	6	6 Slavonia	6 Unknown	6 Unknown	6 Ocular	6 Unkonown	6 *D. repens*
[23]	2016	1	Central Croatia	Domestic	No	Genital	Scrotum	*D.repens*
[24]	2018	1	Dalmatia	No	Dog	Ocular	Subconjunctival	*D. repens*
[25]	2019	1	Central Croatia	No	Dog and domestic animals	Genital	Pubic region	*D. repens*
⁎	2020	3	3 Istria	2 Domestic 1 No	1 Dog and cat 1 Cat 1 No	2 Subcutaneous 1 Ocular	2 Upperarm 1 Eyelid	3 *D. repens*

⁎ Current study.

## 4 Discussion

Human dirofilariasis is an endemic disease that is historically linked to the Mediterranean area but today, due to the increase in its prevalence, more and more countries are considered endemic areas [5,36]. Ongoing climate changes (global warming) and global movement are considered the most important factors for dirofilariasis expansion, both in terms of prevalence and the geographical spread. The emergence of human dirofilariasis correlates with the expansion of competent vector species and increased parasite survival in certain areas [[36], [37], [38]].

In Croatia, *D. repens* is an established parasite both in animals and humans [5]. Since the first description of the human case in a patient with traveling history in 1996, the first autochthonous case was confirmed seven years after. A total of 30 cases of human dirofilariasis caused by *D. repens* have been described in Croatia since these first reports. While the true prevalence of dirofilariasis of Croatia is unknown, due to a lack of mandatory reporting, it is nevertheless interesting to speculate on the epidemiology of this infectious disease in this country [39]. In Ukraine, introduction of mandatory reporting system of *D. repens* human infection as notifiable disease in 1975 enabled surveillance and establishment of proper diagnostics. National register has resulted in high accuracy (75%) of primary care physicians in setting the correct diagnosis, evaluation of the disease spread from south-eastern to north-western regions and increased incidence rate [40]. Until 2007, all cases in Croatia were reported only from the coastal region. Since then, the number of cases in the continental region have continuously increased with the geographical distribution now appearing almost identical. The possible explanation for the emergence of human dirofilariasis in the continental region of Croatia is the expansion of the host range of the competent vector species, *Aedes albopictus* and *Aedes japonicus* [41]. The first reports of human cases in Croatia coincide with *A. albopictus* detection in Istria and, later, in most of the Dalmatian areas and islands, as well as in Croatia's capital, Zagreb [26,42,43]. Invasive and locally highly abundant mosquitoes are well known for their contribution to local transmission of filarial worms [44]. The occurrence of human dirofilariasis depends on the availability of susceptible vector species and the climate suitable for successful intra-mosquito development [36]. Although dogs represent the most important reservoirs, we believe that the mosquito population is more important for emergence of human cases [33,35]. In the region of Daruvar (Central Croatia) (latitude 45.3524° N; longitude 17.1348° E), the prevalence of dirofilariosis in dogs is around 45% and human cases are still lacking [33]. Contrary to that, after extensive flooding in Gunja (Slavonia) (latitude 44.5248° N; longitude 18.5100° E) in 2014, a small cluster of six cases of dirofilariosis was reported from the affected region, possibly as a result of increased mosquito activity [22]. After floods, floodwater mosquito species are characterized with increased vectorial performance, especially a wider geographical distribution, shorter development cycle, polycyclicity, ability to form multiple populations, the ability of females to fly to longer distances from the reproduction site and wider host preference [45,46]. Interestingly, all six cases from the flooded region were of ocular dirofilariasis [22].

Clinical data from the current study showed that, consistent with previous cases described from the bibliography of Croatian dirofilariasis reports, *D. repens* had a typical predilection for subcutaneous and ocular tissues. Reviewing more than 1700 human cases from world literature, Simeon et al. reported subcutaneous/ocular dirofilariasis in approximately 80% of cases [1]. However, this review included cases of *D. immitis* infection, which is typically associated with pulmonary dirofilariasis. *D. immitis* has never been detected as causative agent of human dirofilariasis in Croatia, although it was detected in dogs in several localities in coastal Croatia [33,47]. Subcutaneous nodules were not the major clincal presentation of *D. repens* infections in our study, despite the fact that this presentation is by far the major clinical presentation of *D. repens* infection, reported in approximately 55–82% of affected individuals [3,6,48,49]. Results from our review of Croatian cases revealed the distribution of infestation among subcutaneous and ocular tissues was almost identical (43% and 40%, respectively) with the parasite having a predilection for anatomical sites other than the eyes and subcutis in >15% of cases. For example, in three male patients, nematodes were found in genitals and, in two females, the mammary and genital region were the affected sites.

## 5 Conclusion

Numerous cases of human dirofilariasis have been recorded in Croatia over the last 25 years. With a lack of epidemiological reporting and surveillance system, the real number of cases is probably higher than published. In addition, an increase in various predisposing factors suggests that *D. repens* will have an increasing prevalence in Croatia. Autochthonous findings of *D. immitis* in dogs and the presence of competent vectors capable of its transmission also point to this nematode becoming a causative agent of human dirofilariasis in Croatia as well. Further dissemination of human dirofilariasis is related to changes in Croatian mosquito fauna, caused by ongoing climate changes. Temperature rise favours shorter development cycles of autochthonous mosquito vectors and expansion of the invasive species *A. albopictus* and *A. japonicus* in Croatia. Evidence of climate change can be seen in the enhanced amount of rainfall which through flooding and increased vector activity caused a human outbreak few months later. The introduction of mandatory reporting system, environmental and public health strategies, raising awareness among health care professionals, screening of blood-fed and host-seeking mosquitoes, and screening and protection of dogs could contribute to the establishment of efficient preventive measures against human dirofilariasis in Croatia. Adopting “One Health” approaches that involve close collaboration between epidemiologists, physicians, veterinarians, public health and environmental experts is essential for a successful response to future challenges presented by this emerging zoonotic agent.

## 6 Ethics declarations

**Ethics approval and consent to participate.**

The authors assert that all procedures contributing to this work are approved and comply with the ethical standards of the relevant national and institutional committees on human experimentation and with the Helsinki Declaration of 1975, as revised in 2008.

**Consent for publication.**

Case reports are retrospective. Only archived patients' data and samples processed for diagnostic purposes were used. Prior the processing, written consent with examination and diagnostic tests, as well as with potential further use of the data and samples for scientific or educational purposes including publication of anonymized data was provided by each particular patient.

## Declarations of interest

None.

## Availability of data and materials

The data generated during this study are included within this manuscript or are available upon request from the corresponding author.

## Financial support

This research did not receive any specific grant from funding agencies in the public, commercial, or not-for-profit sectors.

## Authors' contributions

APB: Conceptualization, Investigation, Writing - Original Draft. JPB: Investigation, Writing - Original Draft, Visualization. DJ: Methodology. MC, LLS, IAC, JK: Resources. AP: Writing - Review & Editing. RB: Conceptualization, Methodology, Validation, Supervision.
